# Bone-derived Nestin-positive mesenchymal stem cells improve cardiac function via recruiting cardiac endothelial cells after myocardial infarction

**DOI:** 10.1186/s13287-019-1217-x

**Published:** 2019-04-27

**Authors:** Dihan Lu, Yan Liao, Shuang-Hua Zhu, Qiao-Chao Chen, Dong-Mei Xie, Jian-Jun Liao, Xia Feng, Mei Hua Jiang, Wen He

**Affiliations:** 1 Department of Anesthesiology, The First Affiliated Hospital, Sun Yat-Sen University, Guangzhou, Guangdong 510080 People’s Republic of China; 2 Key Laboratory for Stem Cells and Tissue Engineering, Center for Stem Cell Biology and Tissue Engineering, Ministry of Education, Sun Yat-Sen University, Guangzhou, Guangdong 510080 People’s Republic of China; 3 Department of Cardiology, the Third Affiliated Hospital, Sun Yat-Sen University, Guangzhou, Guangdong 510080 People’s Republic of China; 4 Department of Geriatrics, The First Affiliated Hospital, Sun Yat-Sen University, Guangzhou, Guangdong 510080 People’s Republic of China; 5 Department of Cardiology, the First Affiliated Hospital, Sun Yat-Sen University, Guangzhou, Guangdong 510080 People’s Republic of China; 6 Department of Anatomy, Zhongshan School of Medicine, Sun Yat-Sen University, Guangzhou, Guangdong 510080 People’s Republic of China

**Keywords:** Nestin, Mesenchymal stem cell, Cortical bone, Chemotaxis, Myocardial infarction

## Abstract

**Background:**

Bone-derived mesenchymal stem cell (BMSC) transplantation has been reported to be effective for the treatment of ischemic heart disease, but whether BMSCs are the optimal cell type remains under debate. Increasing numbers of studies have shown that Nestin, an intermediate filament protein, is a potential marker for MSCs, which raises the question of whether Nestin^+^ cells in BMSCs may play a more crucial role in myocardial repair**.**

**Methods:**

Nestin^+^ cells were isolated using flow cytometry by gating for CD45^−^ Ter119^−^ CD31^−^ cells from the compact bone of Nestin-GFP transgenic mice, expressing GFP driven by the Nestin promoter. Colony-forming and proliferative curve assays were conducted to determine the proliferative capacity of these cells, while qRT-PCR was used to analyze the mRNA levels of relative chemokines and growth factors. Cardiac endothelial cell (CEC) recruitment was assessed via a transwell assay. Moreover, permanent ligation of the left anterior descending (LAD) coronary artery was performed to establish an acute myocardial infarction (AMI) mouse model. After cell transplantation, conventional echocardiography was conducted 1 and 4 weeks post-MI, and hearts were harvested for hematoxylin-and-eosin (HE) staining and immunofluorescence staining 1 week post-MI. Further evaluation of paracrine factor levels and administration of a neutralizing antibody (TIMP-1, TIMP-2, and CXCL12) or a CXCR4 antagonist (AMD3100) in MI hearts were performed to elucidate the mechanism involved in the chemotactic effect of Nestin^+^ BMSCs in vivo.

**Results:**

Compared with Nestin^−^ BMSCs, a greater proliferative capacity of Nestin^+^ BMSCs was observed, which further exhibited moderately high expression of chemokines instead of growth factors. More CEC recruitment in the Nestin^+^ BMSC-cocultured group was observed in vitro, while this effect was obviously abolished after treatment with neutralizing antibodies against TIMP-1, TIMP-2, or CXCL12, and more importantly, blocking the CXCL12/CXCR4 axis with a AMD3100 significantly reduced the chemotactic effect of Nestin^+^ BMSCs. After transplantation into mice exposed to myocardial infarction (MI), Nestin^+^ BMSC-treated mice showed significantly improved survival and left ventricular function compared with Nestin^−^ BMSC-treated mice. Moreover, endogenous CECs were markedly increased, and chemokine levels were significantly higher, in the infarcted border zone with Nestin^+^ BMSC treatment. Meanwhile, neutralization of each TIMP-1, TIMP-2, or CXCL12 in vivo could reduce the left ventricular function at 1 and 4 weeks post-MI; importantly, the combined use of these three neutralizing antibodies could make a higher significance on cardiac function. Finally, blocking the CXCL12/CXCR4 axis with AMD3100 significantly reduced the left ventricular function and greatly inhibited Nestin^+^ BMSC-induced CEC chemotaxis in vivo.

**Conclusions:**

These results suggest that Nestin^+^ BMSC transplantation can improve cardiac function in an AMI model by recruiting resident CECs to the infarcted border region via the CXCL12/CXCR4 chemokine pathway. And we demonstrated that Nestin^+^BMSC-secreted TIMP-1/2 enhances CXCL12(SDF1α)/CXCR4 axis-driven migration of endogenous Sca-1^+^ endothelial cells in ischemic heart post-AMI. Taken together, our results show that Nestin is a useful marker for the identification of functional BMSCs and indicate that Nestin^+^ BMSCs could be a better therapeutic candidate for cardiac repair.

**Electronic supplementary material:**

The online version of this article (10.1186/s13287-019-1217-x) contains supplementary material, which is available to authorized users.

## Introduction

Acute myocardial infarction (MI) is a common disease that is followed by the necrosis of millions of cardiac cells, resulting in the replacement of the myocardium with a dense fibrotic scar and ventricular remodeling [1], thereby critically endangering the heart’s functional performance. Stem cell therapy has been demonstrated to replenish damaged cells and to lead to successful cardiac regeneration. This therapy includes repair using autologous cardiac [2–4] and bone marrow-derived [5, 6] stem cells and the reprogramming of endogenous non-stem cells into cardiac cells [7]. Mesenchymal stem cells (MSCs) are among the most frequently investigated cellular populations. The results from some preclinical studies and clinical trials have demonstrated that the transplantation of bone marrow-derived stem cells (BmMSCs) into the ischemic region of an MI heart can improve cardiac functional recovery [8, 9] and reverse adverse cardiac remodeling through differentiation of BmMSCs into functional cardiomyocytes [10], paracrine signaling [11], and stimulation of endogenous cardiac progenitor cell (CPC) proliferation [6].

Although the bone marrow is the most common source of MSCs, recent studies have revealed the inconsistent effects of using BmMSCs as a cell source of transplantation therapy, as they have the risk of hematopoietic cell contamination [12, 13]. Therefore, searching for an alternative source of MSCs with better regenerative capacity for tissue repair is currently underway. Bone-derived mesenchymal stem cell (BMSC) transplantation therapy has been tested in a mouse model of MI and has been demonstrated to increase the survival rate, improve cardiac function, and attenuate remodeling via secreted factors, which stimulate endogenous neovascularization and differentiation into functional adult myocytes and vascular cells [14, 15]. However, whether there is a subpopulation acting as major functional cells to participate in tissue repair among the BMSCs remains under debate.

Nestin, widely known as a neural stem cell marker, is a member of the intermediate filament protein family and is also expressed in stem/progenitor cell populations, indicating that it might be a potential indicator of proliferative and multipotent progenitor cells [16]. Several types of stem cells have been identified using Nestin as a tracking marker, including BmMSCs [17], hair follicle sheath progenitor cells [18], endothelial progenitor cells [19], and testicular stem Leydig cells [20]. Emerging evidence suggests that Nestin^+^ cells in the hematopoietic stem cell (HSC) niche, believed to be MSCs, constitute an essential HSC niche component [17], which raises the question of whether Nestin^+^ cells in BMSCs play a more crucial role in tissue repair.

In this study, we isolated Nestin^+^ and Nestin^−^ BMSCs from Nestin-GFP transgenic mice by flow cytometry and further verified the proliferation and multilineage differentiation capacity of both groups. Thereafter, related paracrine factors (growth factors and chemokines) were analyzed by quantitative real-time polymerase chain reaction assays (qRT-PCR). A transwell migration assay was also performed, which showed a higher chemokine secretion level and more cardiac endothelial cells (CEC) migration with an enhanced chemotactic effect in the Nestin^+^ BMSC-cocultured group. To further evaluate the role of the cells in restoring cardiac function, cells were transplanted to a damaged heart post-MI, where Nestin^+^ BMSCs significantly improved cardiac function compared to Nestin^−^ BMSCs. Finally, histological analyses were conducted to investigate the underlying mechanisms participating in the therapeutic effects of Nestin^+^ BMSCs post-MI.

## Materials and methods

### Mice

Homozygous Nestin-GFP transgenic mice that express enhanced GFP under the control of the Nestin promoter (Nes-GFP), which were on the C57BL/6 genetic background, were kindly provided by Dr. Masahiro Yamaguchi as previously reported [21]. C57BL/6 mice were purchased from the Experimental Animal Center of Sun Yat-sen University (Guangzhou, China). All animal protocols were reviewed and approved by the Sun Yat-Sen University Institutional Animal Care and Use Committee.

### Bone-derived Nestin^+^ cell isolation and culturing

MSCs from mouse compact bones were harvested as previously reported [22]. Seven-day-old Nes-GFP or C57BL/6 mice (blank control) were sacrificed under deep general anesthesia, then femurs and tibiae were harvested. After bone marrow cavities were flushed at least six times, the collected bones were minced followed with flushing out and incubated with 20 ml Hanks’ balanced salt solution (HBSS) digestive solution including collagenase II (300 U/ml; Gibco, Grand Island, NY, USA). After tissue homogenization, the small tissues were incubated at 37 °C for 60 min with shaking at 200 r/min. Subsequently, the cell suspensions were passed through a 40-μm cell strainer, yielding single cells. The GFP-positive and GFP-negative cells were sorted using flow cytometry (Influx, BD, NJ, USA) by gating for CD45^−^ Ter119^−^ CD31^−^ population and cultured on plastic plates in L-Dulbecco’s modified essential medium (DMEM) with 10% fetal bovine serum (FBS) (Gibco). The cells were then isolated by their characteristic of plastic adherence by changing the supernatant 6 h after primary seeding, and were cultured at 37 °C under 5% CO_2_ while being propagated every 3 days. To expand, the adherent cells were passaged by 0.125% trypsin/0.008% ethylenediamine tetraacetic acid (EDTA).

### Primary cardiac Sca-1^+^ cell isolation and culturing

Seven-day-old C57BL/6 mice hearts were harvested. The collected hearts were minced and incubated with 5 ml HBSS digestive solution including Type II collagenase (300 U/ml; Gibco) and DNase I (100 U/ml; Sigma-Aldrich, St. Louis., MO, USA) at 37 °C for 30 min with shaking every 10 min. Subsequently, the cell suspensions were passed through a 40-μm cell strainer to yield single cells and resuspended with HBSS. Sca-1^+^ cells were sorted via fluorescence-activated cell sorting (FACS) after being incubated with a primary antibody (1:100) for 30 min. Cells were then cultured in DMEM/F12 (Hyclone, Logan, UT, USA) mixed medium with 10% FBS, 10 ng/ml epidermal growth factor (EGF) (Peprotech, Rocky Hill, NJ, USA), 10 ng/ml basic fibroblast growth factor (bFGF) (Peprotech), 10 ng/ml insulin-like growth factor (IGF) (Peprotech), 10 ng/ml leukemia-inhibitory factor (LIF) (Sigma-Aldrich), 1% L-glutamine (Sigma-Aldrich), and 1% penicillin/streptomycin (Invitrogen, Carlsbad, California, USA) as previously reported [23]. The cells were cultured at 37 °C under 5% CO_2_ and propagated every 3 days.

### Primary bone marrow-derived stem cell isolation and culturing

Mouse BmMSCs were isolated from the bone marrow of 7-day-old Nes-GFP mice according to the improved low-density culture method reported previously [24]. Briefly, femurs and tibiae were removed and placed on ice in 5 ml L-DMEM complete medium. Each bone marrow cavity was flushed with the medium, and individual cells were obtained by filtration through a 70-μm cell strainer. After red blood cells were removed by ammonium chloride lysis, the remaining cells were washed with HBSS. GFP-positive cells were sorted using flow cytometry (Influx, BD) and cultured on plastic plates in L-DMEM complete medium, and plated in culture flasks at the low density of 5 × 10^4^ cells/cm^2^. The cells were then cultured for 3 days, and nonadherent cells were removed by a complete change of the medium, while the remaining adherent cells were cultured continuously.

### Flow cytometry analysis

Flow cytometry sorting and flow cytometry analysis were performed with Influx (BD, USA) and a Gallios flow cytometer (Beckman Coulter, Fullerton, CA, USA) respectively. Data were analyzed with FlowJo7.5 software (Treestar, Ashland, OR, USA) or Kaluza software (Beckman Coulter). The following anti-mouse antibodies were used: CD44-PE (IM7), CD105-Alexa Fluor 647 (429), CD31-APC (390), c-Kit-APC-eFluor® 780 (2B8), CD45-PE-Cyanine7 (30-F11), CD11b-APC (M1/70), CD184(CXCR4)-APC (2B11), and F4/80-eFluor 450 (BM8), which were purchased from eBioscience (Hatfield, UK); Sca-1-V450 (D7) was purchased form BD Biosciences (San Diego, CA, USA).

### Colony-forming unit-fibroblast (CFU-F) assay

To perform the CFU-F assay, dissociated single-cell suspensions of cells were diluted to a density of 500 cells/ml, after which 2 μl/well of the diluted cell suspension was plated in 96-well plates; 150 μl of expansion medium was added to each well. Cells were cultured for 10 days; the medium was changed every 3 days. The plates were then washed and stained with 1% (*w*/*v*) crystal violet (Sigma-Aldrich) at room temperature for 5 min. All colonies containing > 50 cells under microscopic observation were counted. The experiment of each group was conducted in triplicate separately, and the spheres were counted by two individuals in a blinded fashion.

### Cell differentiation assay

For osteogenic, adipogenic, and chondrogenic differentiation, cells were cultured in conditioned differentiation mediums for 2–3 weeks and analyzed by staining with Alizarin Red, Oil Red O, and Van Gieson respectively as reported before [24]. Meanwhile, some differentiation-related genes were analyzed by qRT-PCR, including osteogenic genes (RunX2, OCN), adipogenic genes (LPL, FabP4, and PPAR-γ), and chondrogenic genes (Collagen II and Collagen X). The experiment of each group was conducted separately, in triplicate wells.

### RNA isolation, reverse transcription, and quantitative real-time PCR (qRT-PCR)

The total RNA of cultured cells was extracted using TRIzol reagent (Invitrogen). Total 1 μg of RNA was reverse transcribed using the RevertAid First Strand cDNA Synthesis Kit (Thermo Scientific, Waltham, MA, USA). The cDNA thus obtained was subjected to real-time PCR with the SYBR Green reagent (Roche, Indianapolis, IN, USA). Quantitative real-time PCR was performed as described elsewhere. Mouse primers used are listed in Table 1. The relative mRNA abundance was calculated using the ∆Ct or ∆∆Ct methods, and gene expression levels were normalized with respect to those of GAPDH.

**Table 1:** Primers used for the amplification of mouse transcripts by quantitative real-time PCR

Genes	Forward sequence	Reverse sequence
GAPDH LPL FabP4 PPAR-γ RunX2 OCN Collagen II Collagen X TGF-β SCF Angpt1 FGF2 FGF7 LIF CTGF VEGF PDGF IGF-1 HGF MCP1 CXCL12 CSF1 TIMP-1 TIMP-2 Collagen III Collagen IV MMP2 MMP9	5′-ACCACAGTCCATGCCATCAC-3′ 5′-GCGTAGCAGGAAGTCTGACCAA-3′ 5′-AATCACCGCAGACGACA-3′ 5′-CTGACCCAATGGTTGCT-3′ 5′-CGTGGCCTTCAAGGTTGTA-3′ 5′-GCAATAAGGTAGTGAACAGACTCC-3′ 5′-GCTGGTGAAGAAGGCAAACGAG-3′ 5′-GTACCAAACGCCCACAGGCATA-3′ 5′-TGATACGCCTGAGTGGCTGTCT-3′ 5′-ATCTGCGGGAATCCTGTGACTG-3′ 5′-AACCGAGCCTACTCACAGTACG-3′ 5′-AAGCGGCTCTACTGCAAGAACG-3′ 5′-TGTTCTGTCGCACCCAGTGGTA-3′ 5′-TCAACTGGCACAGCTCAATGGC-3′ 5′-TGCGAAGCTGACCTGGAGGAAA-3′ 5′-CTGCTGTAACGATGAAGCCCTG-3′ 5′-AATGCTGAGCGACCACTCCATC-3′ 5′-GTGGATGCTCTTCAGTTCGTGTG-3′ 5′-GTCCTGAAGGCTCAGACTTGGT-3′ 5′-GCTACAAGAGGATCACCAGCAG-3′ 5′-GGAGGATAGATGTGCTCTGGAAC-3′ 5′-GCCTCCTGTTCTACAAGTGGAAG-3′ 5′-TCTTGGTTCCCTGGCGTACTCT-3′ 5′-AGCCAAAGCAGTGAGCGAGAAG-3′ 5′-GACCAAAAGGTGATGCTGGACAG-3′ 5′-ATGGCTTGCCTGGAGAGATAGG-3′ 5′-CAAGGATGGACTCCTGGCACAT-3′ 5′-GCTGACTACGATAAGGACGGCA-3′	5′-TCCACCACCCTGTTGCTGTA-3′ 5′-AGCGTCATCAGGAGAAAGGCGA-3′ 5′-GTGGAAGTCACGCCTTTC-3′ 5′-CAGACTCGGCACTCAATG-3′ 5′-GCCCACAAATCTCAGATCGT-3′ 5′-AGATGCGTCTGTAGGCGGTCTT-3′ 5′-CCATCTTGACCTGGGAATCCAC-3′ 5′-GGACCAGGAATGCCTTGTTCTC-3′ 5′-CACAAGAGCAGTGAGCGCTGAA-3′ 5′-CCATATCTCGTAGCCAACAATGAC-3′ 5′-GCATCCTTCGTGCTGAAATCGG-3′ 5′-CCTTGATAGACACAACTCCTCTC-3′ 5′-TTCCAACTGCCACGGTCCTGAT-3′ 5′-GGAAGTCTGTCATGTTAGGCGC-3′ 5′-CCGCAGAACTTAGCCCTGTATG-3′ 5′-GCTGTAGGAAGCTCATCTCTCC-3′ 5′-TCGGGTCATGTTCAAGTCCAGC-3′ 5′-TCCAGTCTCCTCAGATCACAGC-3′ 5′- CCAGCCGTAAATACTGCAAGTGG-3′ 5′-GTCTGGACCCATTCCTTCTTGG-3′ 5′-AGTGAGGATGGAGACCGTGGTG-3′ 5′-ACTGGCAGTTCCACCTGTCTGT-3′ 5′-GTGAGTGTCACTCTCCAGTTTGC-3′ 5′-GCCGTGTAGATAAACTCGATGTC-3′ 5′-CAAGACCTCGTGCTCCAGTTAG-3′ 5′-TGGTTGCCCTTTGAGTCCTGGA-3′ 5′-TACTCGCCATCAGCGTTCCCAT-3′ 5′- TAGTGGTGCAGGCAGAGTAGGA-3′

### Western blot analysis

Total proteins were extracted, and the protein concentration was measured using a BCA protein assay kit (Thermo Scientific, Rockford, AL, USA). Proteins were separated using 8% or 10% sulfate-poly-acrylamide gel electrophoresis and were then transferred to a polyvinylidene fluoride (PVDF) membrane; the membrane was then blocked with Tris-buffered saline (TBS)/T containing 5% nonfat dry milk and analyzed for the target proteins. The specific antibodies that were used recognized as MCP-1 (E9R7Z), SDF1/CXCL12 (D32F9), and TIMP-2 (D18B7) from Cell Signaling Technology, and TIMP-1 (Clone #151624) and CSF-1 (Clone # 131614) from R&D Systems.

### Transwell migration assay

Cultured Nestin^+^ and Nestin^−^ BMSCs were seeded on the lower chamber of the 24-well plates, while Sca-1^+^ CECs were plated at 5 × 10^4^ cells/well on the upper compartment of 24-well transwell inserts (8-mm pore size insert, Millipore, Billerica, MA, USA), and the plates were then incubated for 12 h at 37 °C under 5% CO_2_. In the control group, no cell was seeded on the lower chamber. After the incubation, the transwell inserts were discarded, and the upper side of the filter was gently swabbed to remove the non-migratory cells. Migrated cells on the lower side of the insert filter were then quickly fixed using 4% paraformaldehyde (PFA) and stained with 0.5% crystal violet for 20 min. Neutralization assays were performed in a transwell system supplemented with anti-TIMP-1 (1.5 μg/ml; R&D Systems), anti-TIMP-2 (3 μg/ml; R&D Systems), and anti-CXCL12 (100 μg/ml; R&D Systems). Microscopic examination was performed and five low-power fields (magnification, × 400) were randomly selected from each chamber. All the experiments in each group were performed in triplicate. The migrated cells were counted by two individuals in a blinded fashion.

### Mouse myocardial infarction, cell transplantation, and echocardiography

The mouse model of acute myocardial infarction (AMI) was performed by permanent ligation of the left anterior descending (LAD) coronary artery. One minute after artery occlusion, the ischemic area was identified and 25 μl of cell suspensions containing 3 × 10^5^ bone-derived Nestin^+^ cells, Nestin^−^ cells, or saline (vehicle control) was intramyocardially injected into the infarcted border zone as previously described [25]. Injected cells were labeled with CellTracker CM-Dil (Vybrant™ Dil cell-labeling solution, Thermo Scientific) (50 μg/ml) 30 min prior to transplantation. In the Nestin^+^ BMSC+AMD3100 treatment group, 5 μl AMD 3100 (200 mmol/L, Selleckchem, Houston, USA) dissolved in saline was intramyocardially injected along with cell treatment. To determine the effect of bone-derived Nestin^+^ cells and Nestin^−^ cells on infarct size and endogenous CEC recruitment, mouse hearts were harvested 1 week after MI for hematoxylin-and-eosin (HE) and immunofluorescence staining. Hearts were fixed and embedded in paraffin and sectioned into 5-μm slices. Conventional echocardiography (C57BL/6 mice from each group at 1 and 4 weeks after MI) was performed with a mouse echocardiography system (Vevo 2100 Imaging System, VisualSonics, Toronto, Canada), equipped with a 30-MHz phased transducer. Experiments were conducted and data were collected by two individuals in a blinded fashion.

### Histological analysis

The paraffin-embedded sections were dewaxed by baking at 60 °C for 45–60 min, cleaned with xylene, and rehydrated with 100% ethanol and 70% ethanol. After being washed in distilled water, the sections were stained in hematoxylin (Sigma-Aldrich) for 2 min and washed two times in running water for 1 min. The sections were dipped in ammonium hydroxide solution and then washed again. After rinsing each section in graded ethanol, 10 dips were performed in Eosin-Y (Sigma-Aldrich) solution for 30–45 s to counterstain the sections. After additional rinses in 100% ethanol, the sections were dehydrated in graded alcohols, cleared in xylene, and cover-slipped.

### Apoptosis analysis

In an in vitro experiment, cardiac muscle HL-1 cells (purchased from ATCC company) were treated without or with 100 μM H_2_O_2_ for 8 h, and then co-cultured with Nestin^+^ or Nestin^−^ BMSCs in a transwell system. After being cocultured for 24 h, the HL-1 cells (at the bottom of transwell system) were collected and resuspended in 100 μl binding buffer. Cell density in the cell suspension was adjusted to 2 × 10^3^ cells/μl. Subsequently, 5 μl Annexin V-FITC was added to the cell suspension followed by gently vortexing and incubation for 10 min at room temperature in the dark. Thereafter, the cell suspension was incubated with 5 μl propidium iodide. All details need to refer to the instructions of the kit (BD Bioscience, San Diego, CA, USA). Cells were analyzed using flow cytometry for Annexin V-FITC and propidium iodide binding immediately.

In an in vivo experiment, TUNEL (transferase-mediated deoxyuridine triphosphate-biotin nick end labeling) staining was performed using an in Situ Cell Death Detection kit, TMR red (Roche Diagnostics GmbH, Germany), as per the manufacturer’s instruction. Briefly, heart tissues were fixed in 4% PFA and tissues were dehydrated in 30% sucrose. The dehydrated hearts were cut into 5-μm sections and DNA fragmentation was assessed. DAPI was used to stain the nuclei, and the cells were imaged using the Zeiss LSM-780 Confocal Microscope (Carl Zeiss, Germany). Apoptosis/necrosis was quantified by the ratio of TUNEL-positive nuclei to total cell nuclei. Microscopic examination was performed and was randomly selected from each section. Each experiment was performed in triplicate for each group.

### Immunofluorescence staining

For immunofluorescence, cells and heart sections were blocked with normal goat serum for 40 min, and then incubated with primary antibodies overnight at 4 °C, followed by incubation with secondary antibodies (1:500 dilution) to protect from the light at room temperature for 1 h. The following primary antibodies were used: anti-Sca-1 (1:100, Santa Cruz), anti-Islet-1 (1:300, Abcam), Nkx2.5 (1:100, Santa Cruz), and GATA4 (1:100, Santa Cruz). The following secondary antibodies were used: Alexa Fluor Chicken-anti-Rat 647, Alexa Fluor Goat-anti-Mouse 488, Alexa Fluor Chicken-anti-Rat 594, and Alexa Fluor Goat-anti-Rabbit 594 (Invitrogen). Microscopic examination was performed and was randomly selected from each chamber. All images were obtained using a LSM-780 confocal microscope and were analyzed using ImageJ software.

### Statistics

All results were performed in at least three independent experiments and expressed as mean ± SEM. Comparisons of means between two groups were performed using Student’s *t* test, while comparisons of three or more groups were made by one-way ANOVA followed by *t* test corrected for multiple comparisons. *p* < 0.05 was considered significant. Analysis and graph were performed using GraphPad Prism software 5.01 (San Diego, CA, USA).

## Results

### Isolation and characterization of Nestin^+^ and Nestin^−^ cells from the compact bone

**Fig. 1:**
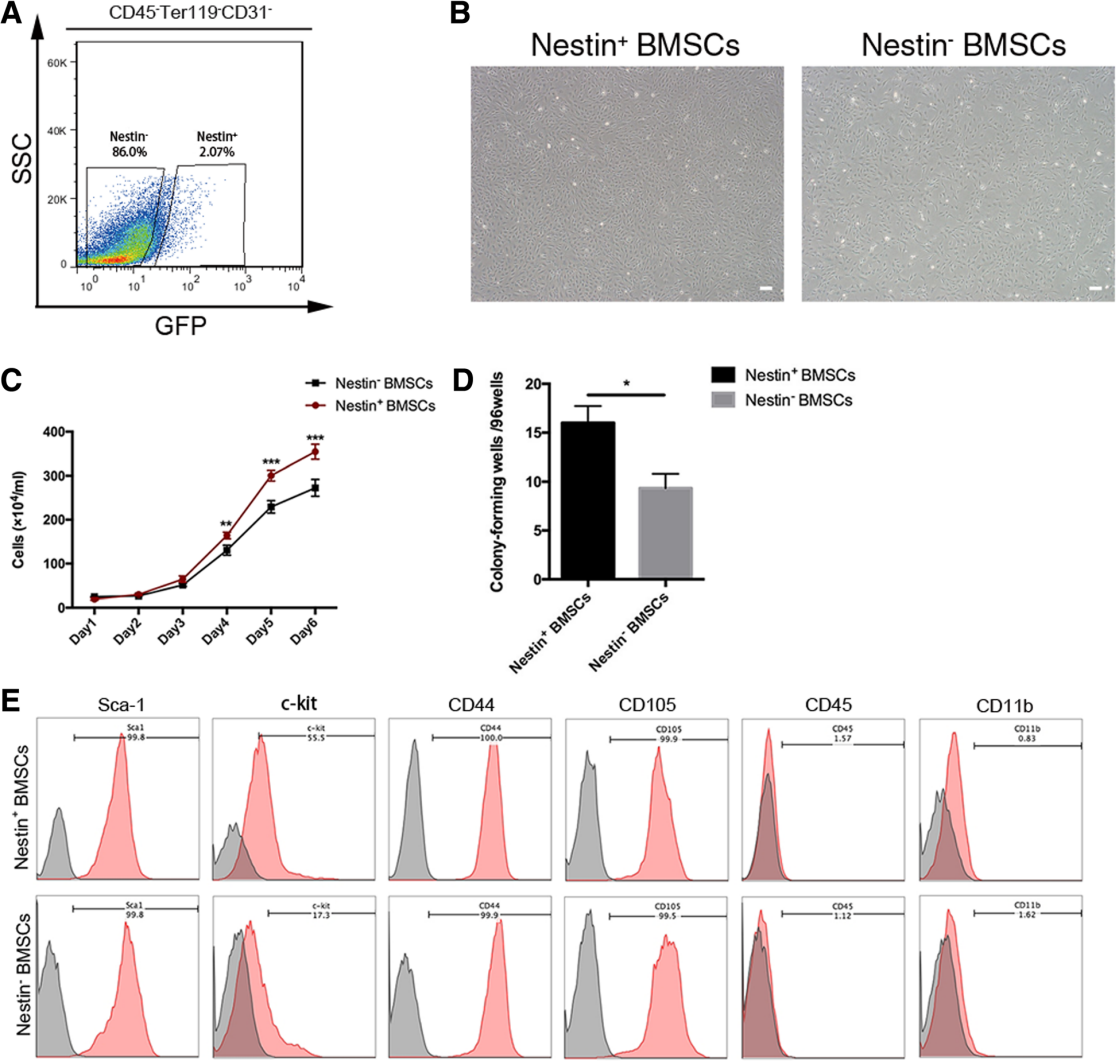
Isolation and proliferation capacity of bone-derived Nestin^+^ and Nestin^−^ cells. **a** Flow cytometry was used to isolate Nestin^+^ and Nestin^−^ cells in the gate of CD45^−^ Ter119^−^ CD31^−^ from the bone of Nestin-GFP transgenic mice. **b** Variations in morphology of the Nestin^+^ and Nestin^−^ cells were captured by microscopy examined at P3. Scale bar, 200 μm. **c** Growth curves of Nestin^+^ and Nestin^−^ cells as assessed by direct counting. Cells at P6 were seeded into a 12-well plate at 10,000 cells/well (triplicates), and the cells were then directly counted for a total of 6 days. **d** Colony-forming unit-fibroblast frequencies of Nestin^+^ and Nestin^−^ cells. Cells at P6 were seeded at a single cell per well into a 96-well plate. Colonies containing > 50 cells were counted under microscopic observation. The means ± SEMs of the results of three different experiments are shown. *p < 0.05; **p < 0.01; ***p < 0.001. **e** Phenotypic characterization of the cultured bone-derived Nestin^+^ and Nestin^−^ cells. Flow cytometry analysis of the presence of the cell surface markers Sca-1, c-kit, CD44, CD105, CD45, and CD11b on cultured bone-derived Nestin^+^ and Nestin^−^ cells

Previous research has suggested that mouse compact bone represents a richer source of MSCs than bone marrow [22]. Additionally, Nestin has been shown to be an indicator of proliferative and multipotent progenitor cells, especially BmMSCs, which suggested that Nestin^+^ BMSCs might be an ideal source for cell transplantation [17]. Toward this end, Nestin^+^ cells were sorted from the compact bones of postnatal day 7 Nestin-GFP transgenic mice or C57BL/6 (as blank control) through FACS by gating for CD45^−^ Ter119^−^ CD31^−^ cells, and Nestin^+^ cells constituted 2.04% ± 0.23% of the total digested compact bone cell population (Fig. 1a).

After primary seeding at a density of 1 × 10^4^/cm^2^, both the Nestin^+^ and Nestin^−^ cell lines were established. The Nestin^−^ cells were clearly sparser under the same culture conditions and magnification at passage 3 (P3) (Fig. 1b). Moreover, the proliferation capacities of Nestin^+^ and Nestin^−^ cells were confirmed by consecutive cell counting for a total of 6 days at P6, which showed the clearly higher proliferation rate of Nestin^+^ cells (Fig. 1c). CFU-F frequencies were further evaluated for the same purpose at P6 and were clearly higher in Nestin^+^ cells (Fig. 1d). These results revealed the greater proliferation capacity of Nestin^+^ cells.

To study the characteristics of Nestin^+^ and Nestin^−^ cells, MSC-specific cell surface markers were detected by flow cytometry analysis (Fig. 1e). The two subtypes of cells shared the same basic panel of markers (Sca-1, c-kit, CD44, CD106, CD90, CD45, and CD11b), whereas Nestin^+^ cells expressed a markedly higher c-kit level (*p* = 0.004). Furthermore, Nestin^+^ and Nestin^−^ cells were both favorable for adipogenic, osteogenic, and chondrogenic activity in a conditioned medium (Additional file 1: Figure S1). Taken together, these results suggest that these Nestin^+^ and Nestin^−^ cells both present stem cell characteristics and could be called BMSCs.

### Nestin^+^ BMSCs expressed higher levels of chemokines and promoted CEC migration in vitro

**Fig. 2:**
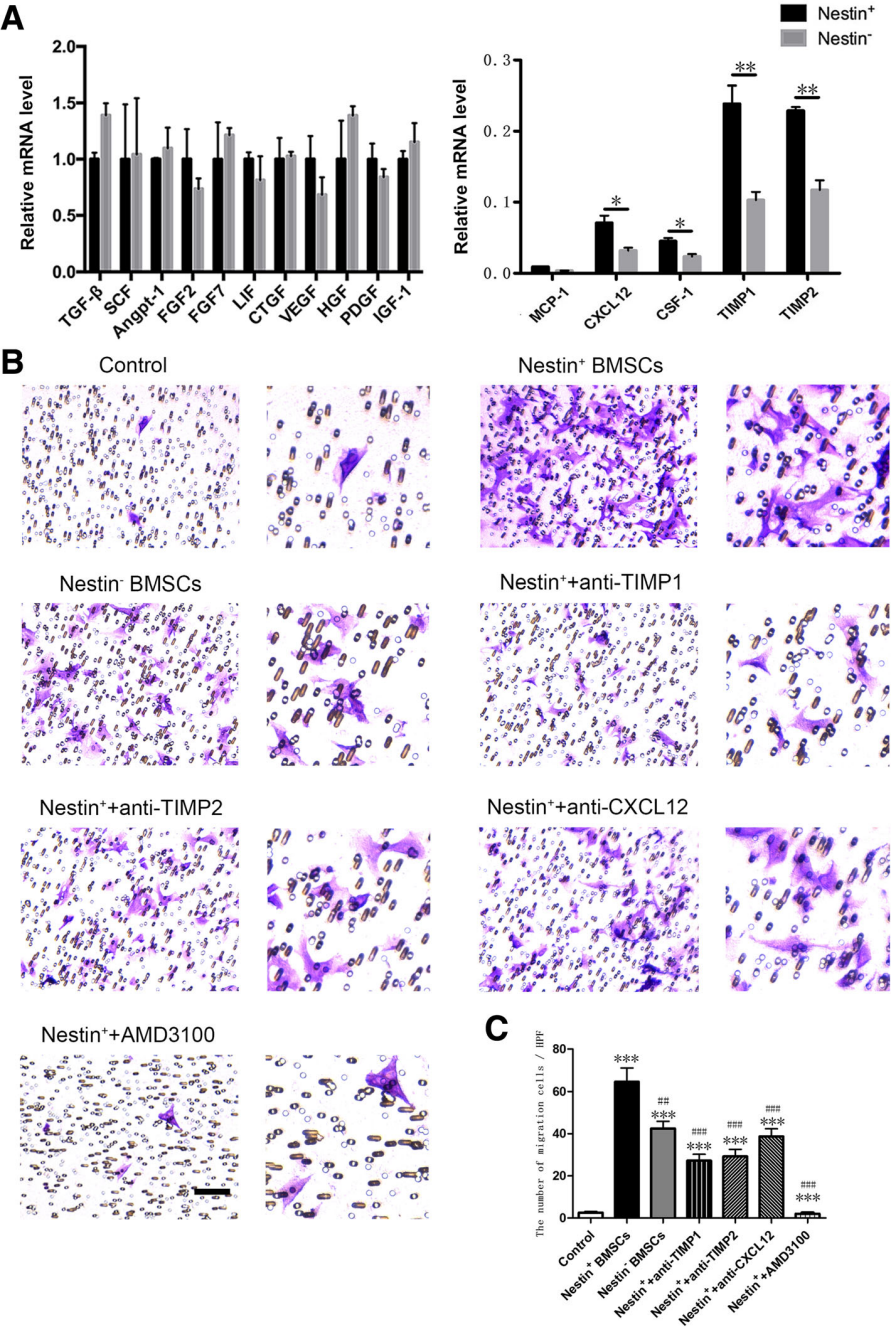
Paracrine factor levels in Nestin^+^ and Nestin^−^ BMSCs and the effect on CEC migration analyzed using transwell migration assay. **a** qRT-PCR analysis of growth factors (TGF-β, SCF-1, Angpt-1, FGF-2, FGF-7, LIF, CTGF, VEGF, HGF, PDGF, and IGF-1) and chemokines (MCP-1, CXCL12, CSF-1, TIMP-1, and TIMP-2) in cultured Nestin^+^ and Nestin^−^ BMSCs relative to GAPDH. Data are shown as the mean ± SEM of triplicate wells in three different experiments. *p < 0.05, **p < 0.01. **b**, **c** Representative images and the number of migrated CECs plotted in different groups. Cells were counted from five different fields for each experiment. The means ± SEMs of the results of triplicate experiments are shown. Scale bar, 100 μm. ***: changed significantly than control group, *p* < 0.001. ##: changed significantly than Nestin^+^ BMSC group, *p* < 0.01; ###, *p* < 0.001

One of the major mechanisms in the repair process using MSCs is paracrine signaling, which includes growth factors, chemokines, cytokines, and survival factors, which might be a way of mediating the process of tissue repair [11, 14, 26]. It was possible that there were differences in the secretion of the paracrine factors between Nestin^+^ and Nestin^−^ BMSCs. The mRNA expression levels of representative growth factors (TGF-β, SCF-1, Angpt-1, FGF2, FGF7, LIF, CTGF, VEGF, HGF, PDGF, and IGF-1) were measured by qRT-PCR analysis, and no difference was found between Nestin^+^ and Nestin^−^ BMSCs (Fig. 2a). In contrast, significantly higher mRNA levels of several representative chemokines (CXCL12, CSF-1, TIMP-1, and TIMP-2) were found in Nestin^+^ BMSCs (Fig. 2a), and the protein level analysis of these chemokines showed that the expression of CXCL12, TIMP-1, and TIMP-2 were significantly higher in Nestin^+^ BMSCs than that in Nestin^−^ BMSCs, but not MCP-1 and CSF-1 (Additional file 2: Figure S2).

Chemokines play a vital role in cardiac progenitor cell (CPC) migration, a process that could relieve tissue damage [27]. To further evaluate the effect of Nestin^+^ and Nestin^−^ BMSCs on CPC migration, Sca-1^+^ cells were isolated from the hearts of postnatal day 7 C57BL/6 mice via FACS and were subsequently cultured (Additional file 3: Figure S3A). After their progenitor and endothelial properties were confirmed (Additional file 3: Figure S3B-E), Sca-1^+^ CECs were seeded on transwell membranes. The results indicated that Nestin^+^ BMSCs attracted markedly more CECs compared to Nestin^−^ BMSCs (Fig. 2b, c), implying a stronger endogenous CEC recruitment effect in vivo. The chemotaxis effects of Nestin^+^ BMSCs on CECs were abrogated obviously when we neutralized TIMP-1, TIMP-2, and CXCL12 chemokines with anti-mouse TIMP-1, TIMP-2, and CXCL12 neutralizing antibodies (Fig. 2b, c). More importantly, Sca-1^+^CD31^+^ CECs also express CXCR4 (Additional file 3: Figure S3F), and AMD3100 significantly reduced the chemotactic effect of Nestin^+^ BMSCs (Fig. 2b, c).

### Transplanted Nestin^+^ BMSCs improve cardiac function post-MI more than Nestin^−^ BMSCs

**Fig. 3:**
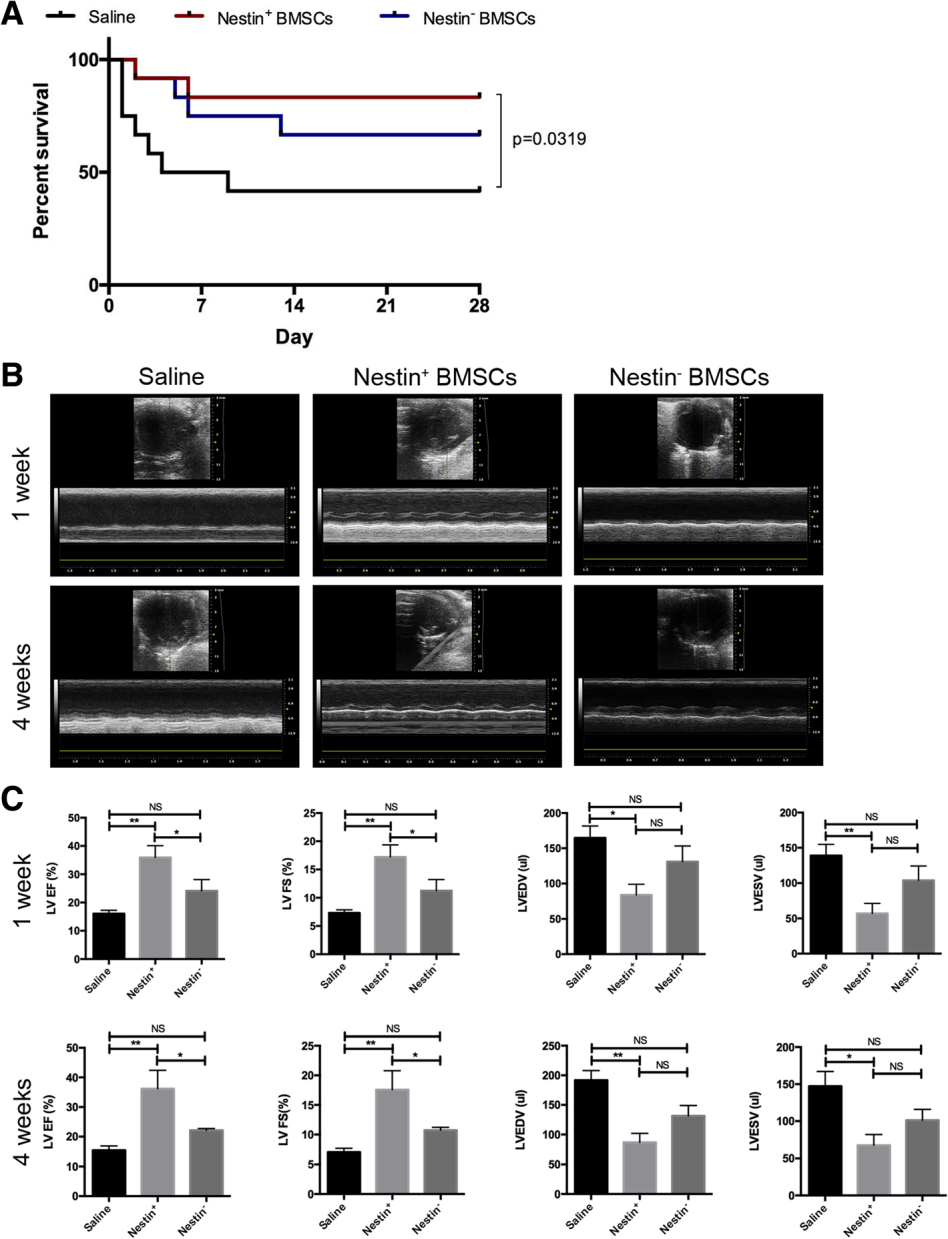
Survival and cardiac functional repair after cell transplantation in mice post-MI. Animals underwent MI + saline, MI + Nestin^+^ BMSC, or MI + Nestin^−^ BMSC surgeries (*n* = 12 for each group) and received follow-up echocardiography in 1 and 4 weeks post-MI. **a** Four-week survival. Data were analyzed using a Kaplan–Meier regression, and significance was determined using the log-rank test. **b** Representative M-mode tracings from mice receiving MI + saline, MI + Nestin^+^ BMSCs, or MI + Nestin^−^ BMSCs at 1 and 4 weeks post-MI. **c** Structural and functional parameters derived from echocardiography measurements. The means ± SEMs of the results are shown. *p < 0.05, **p < 0.01. MI, myocardial infarction. LVEF, left ventricular ejection factor. LVFS, left ventricular fractional shortening. LVEDV, left ventricular end-diastolic volume. LVESV, left ventricular end-systolic volume

Intramyocardial stem cell transplantation after MI has already been reported to improve cardiac function and reverse the remodeling of the infarcted region [4]. To investigate the role of Nestin^+^ and Nestin^−^ BMSCs in myocardium repair post-MI, C57BL/6 mice were randomly divided into three groups, a saline control group, a Nestin^+^ BMSC group, and a Nestin^−^ BMSC transplantation group, and underwent permanent ligation of the LAD coronary artery. The MI procedure reduced 4-week survival to 41.9% in mice receiving saline injections. Mice receiving MI and Nestin^+^ BMSCs demonstrated an 83.3% 4-week survival, which was greater than that in the Nestin^−^ BMSC group (66.67%) (Fig. 3a). Echocardiographic measurement was performed at 1 and 4 weeks post-MI to investigate the change in left ventricular (LV) function. Compared with the saline group, where wall-motion abnormalities and progressive ventricle wall thinning were shown by M-mode echocardiograms, Nestin^−^ BMSCs only slightly attenuated the adverse cardiac remodeling. Notably, markedly better cardiac function was observed in the Nestin^+^ BMSC group compared to the Nestin^−^ BMSC group (Fig. 3b). Moreover, the ejection fraction (EF) and fractional shortening (FS) increased by approximately twofold after Nestin^+^ BMSC transplantation compared with saline. These changes were sustained from 1 week to 4 weeks post-MI, while no significant difference was observed in the Nestin^−^ group. In accordance with the results above, the therapeutic effect was most pronounced for the Nestin^+^ BMSC treatment, which showed a marked reduction in LV end-diastolic and systolic volumes (LVEDV and LVESV) at each time point (Fig. 3c).

Whether Nestin^+^ BMSCs or BmMSCs are therapeutically better in cardiac repair was still uncertain. To evaluate the therapeutic function of Nestin^+^ BMSCs compared with Nestin^+^ BmMSCs, we further isolated and characterized Nestin^+^ BmMSCs (Additional file 4: Figure S4A-C) and transplanted these cells to mice exposed to MI following the procedures previously described. The results showed an apparently improved ventricular function index in both the Nestin^+^ BMSC and Nestin^+^ BmMSC groups 1 week and 4 weeks post-MI, yet the differences were not pronounced between the Nestin^+^ BMSC group compared with the Nestin^+^ BmMSC group (Additional file 4: Figure S4D, E).

These results suggested that Nestin^+^ BMSCs had a better performance in restoring cardiac function than Nestin^−^ BMSCs, but the underlying mechanisms of this improvement need further study.

### Nestin^+^ BMSC transplantation enhances endogenous CEC recruitment to the infarct area

**Fig. 4:**
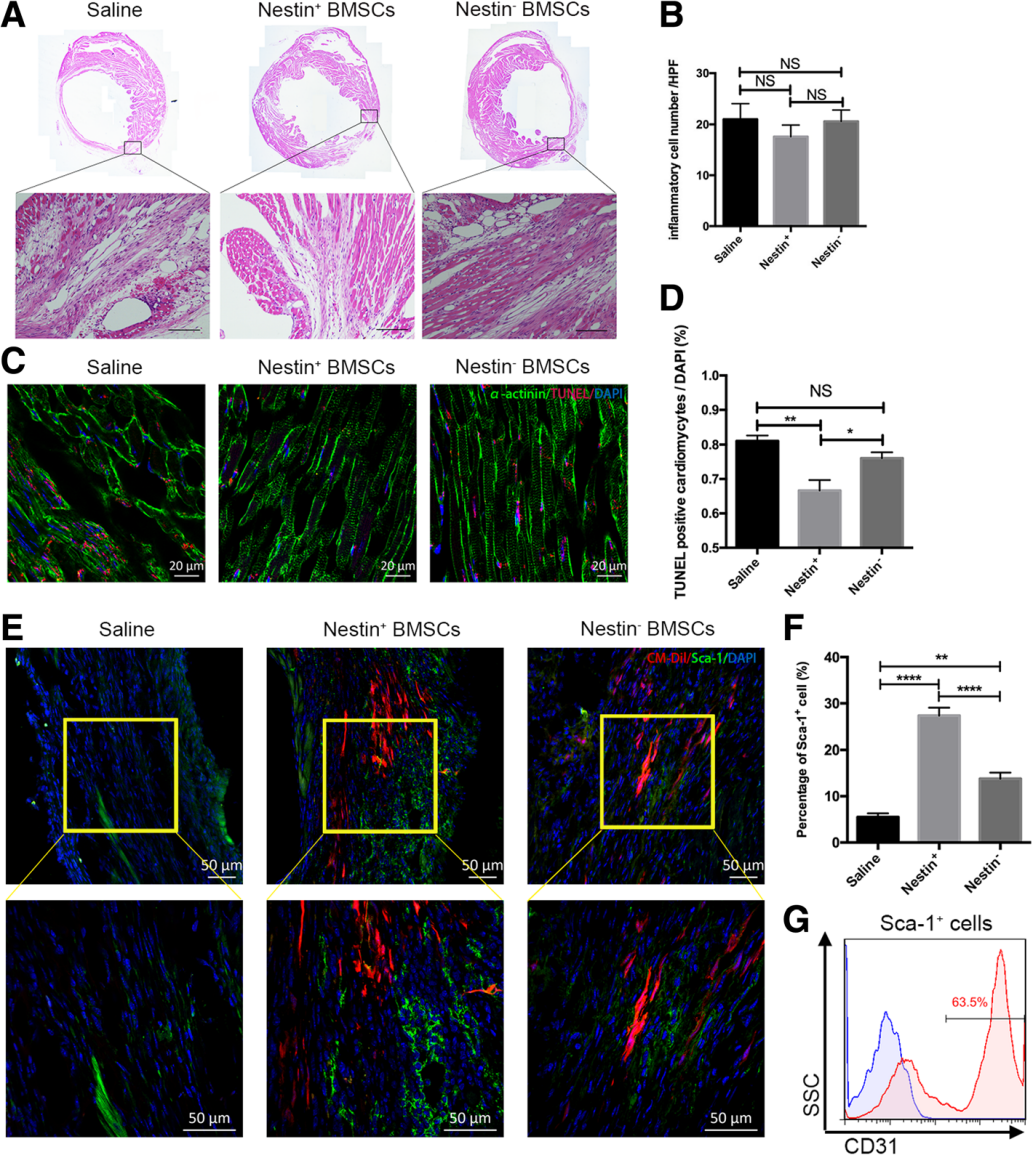
Effects of transplanted Nestin^+^ and Nestin^−^ BMSCs on cardiac remodeling after MI. **a** HE staining of hearts showing inflammatory cells infiltration in different groups (*n* = 3 for each group). Scale bar, 100 μm. **b** The number of inflammatory cells showed no difference among the three groups. Cells were calculated from five randomly different fields under microscopic observation (× 400). Data are shown as the mean ± SEM of triplicate experiments. **c** TUNEL staining of infarcted zone showing the changes in apoptosis cells in different groups (*n* = 3). Scale bar, 20 μm. **d** The number of apoptosis cells showing the reduction of TUNEL-positive cells in Nestin^+^ BMSC group. Data are shown as the mean ± SEM from five different fields. **e** Immunofluorescence staining of cells positive for Sca-1 (green), CM-Dil (red), and DAPI (blue) in the post-MI myocardium after Nestin^+^ or Nestin^−^ BMSC transplantation (*n* = 3 for each group). Scale bar, 50 μm. **f** Percentage of Sca-1-positive cells showing the accumulation of Sca-1^+^ cells in Nestin^+^ BMSC group. Data are shown as the mean ± SEM from five different fields. *p < 0.05, **p < 0.01, ****p < 0.0001. **g** The percentage of CD31 expression in Sca-1^+^ cells, which were isolated from the infarcted heart in the Nestin^+^ BMSC-treated group

To verify the underlying mechanisms involved in myocardium repair after Nestin^+^ BMSC treatment, hearts were harvested at 1 week after MI, and tissue sections were analyzed via HE staining, TUNEL staining, and immunofluorescence staining. Infiltrated inflammatory cells were seen in the infarcted border zone and exhibited no differences among the three groups (Fig. 4a, b). To investigate the apoptosis status of the damaged heart, cell death in the infarcted zone was further evaluated to determine if the Nestin^+^ BMSCs were able to salvage more tissue, which may lead to the preservation of heart function. The results showed a significant reduction of TUNEL-positive cardiomyocytes in the Nestin^+^ BMSC group compared to that in the saline control group, which was more remarkable than the reduction observed in the Nestin^−^ BMSC group (Fig. 4c, d). In keeping with the in vivo results above, in vitro apoptosis assays confirmed the superior protection effect against H_2_O_2_-induced injury of cardiac muscle HL-1 cells in the Nestin^+^ BMSC group relative to the Nestin^−^ BMSC group (Additional file 5: Figure S5). Whether chemotaxis participated in cardiac progenitor cell recruitment in the Nestin^+^ BMSC group, as indicated by the transwell assay, warrants further study. Sca-1^+^ cells are an important CPC type proven to result in endogenous regeneration and cardiac functional recovery repair [28]. Immunofluorescence staining further demonstrated that CM-Dil-stained Nestin^+^ and Nestin^−^ BMSCs were present in the infarcted heart as well as a greater number of Sca-1^+^ cells in the infarcted border zone in the Nestin^+^ BMSC group (Fig. 4e, f), and these recruited endogenous Sca-1^+^ cells exhibited higher CD31 expression, an endothelial cell marker (Fig. 4g).

### Nestin^+^ BMSC-secreted TIMP-1/2 enhances CXCL12(SDF1α)/CXCR4 axis-driven cardiac repair post-AMI

**Fig. 5:**
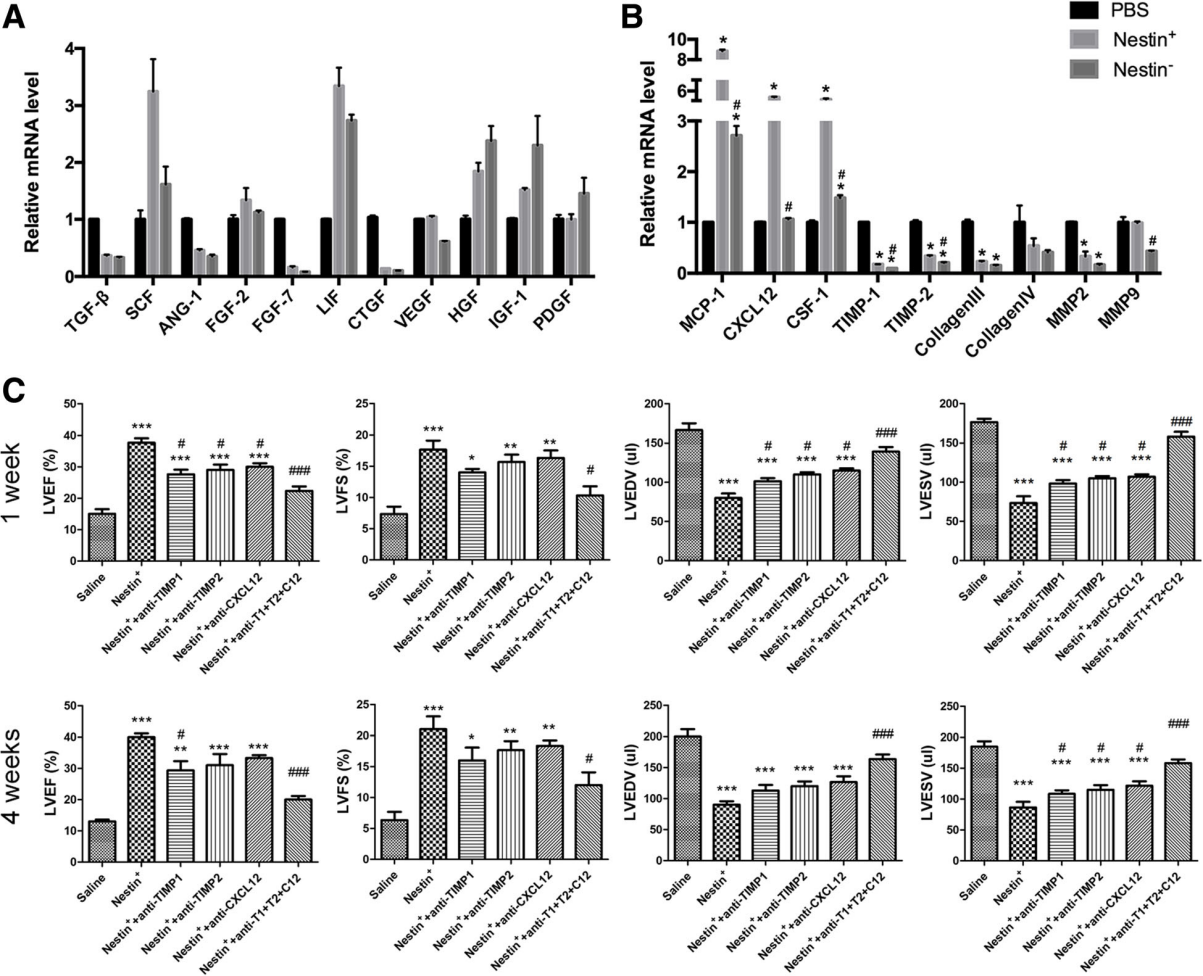
Neutralization of TIPM-1, TIMP-2, and CXCL12 attenuated Nestin^+^ BMSC-mediated restoration of cardiac function after MI. **a** qRT-PCR analysis of growth factors (TGF-β, SCF-1, Ang-1, FGF-2, FGF-7, LIF, CTGF, VEGF, HGF, PDGF, and IGF-1) and **b** chemokines (MCP-1, CXCL12, CSF-1, TIMP-1, TIMP-2, Collagen III, Collagen IV, MMP2, and MMP9) in the myocardial infarcted zone of different groups relative to GAPDH. Data are shown as the mean ± SEM of triplicate wells in three different experiments. *: changed significantly than the PBS group, *p* < 0.05. #: changed significantly than the Nestin^+^ group, *p* < 0.05. **c** Structural and functional parameters derived from echocardiography measurements at 1 and 4 weeks with or without neutralizing antibody treatment. The means ± SEMs of the results are shown. ***: changed significantly than control group, *p* < 0.001; **p < 0.01; *p < 0.05. ##: changed significantly than Nestin^+^ BMSC group, *p* < 0.01; ###, *p* < 0.001; #, *p* < 0.05

To further verify the specific mechanism underlying the enhanced chemotactic effect after Nestin^+^ BMSC transplantation, the growth factors and chemokine levels of in the infarcted heart 1-week post-MI were analyzed by qPCR. In accordance with the previous results in Fig. 2a, the representative growth factor levels were not obviously changed between the Nestin^+^ and Nestin^−^ BMSC transplantation groups (Fig. 5a). Importantly, the chemokine level was relatively higher in the Nestin^+^ BMSC group compared to that in the Nestin^−^ BMSC group, especially the levels of CXCL12, TIMP-1, and TIMP-2 (Fig. 5b). Relevant biomarkers linked with cardiac remodeling (Collagen III, Collagen IV, MMP2, MMP9) have no marked difference between the Nestin^+^ and Nestin^−^ BMSC groups (Fig. 5b).

As elevated chemokine levels (CXCL12, TIMP-1, and TIMP-2) were found in the Nestin^+^ BMSC group both in vivo and in vitro, it was more reasonable to explore the specific mechanism underlying these levels. We found, compared with the Nestin^+^ BMSC treatment group, the neutralization of each TIMP-1, TIMP-2, and CXCL12 chemokines with their specific neutralizing antibody in vivo could obviously reduce the LVEF and LVFS of the MI mice and increase the LVEDV and LVEDS; more importantly, the combined use of these three neutralizing antibodies could make a higher significance between these two groups (Nestin^+^ group vs Nestin^+^+anti-T1+T2+C12 group, Fig. 5c). AMD3100 (an antagonist of CXCR4) was used to block the CXCL12/CXCR4 pathway, which has been reported to participate in the mobilization of CSCs in the heart [29]. Notably, cardiac function measured by M-mode echocardiograms suggested a significantly reduced LV function in the Nestin^+^ BMSC+AMD3100 group relative to the Nestin^+^ BMSC transplantation group (Additional file 6: Figure S6A). Similarly, cotreatment with AMD3100 resulted in an obviously decreased number of Sca-1^+^ CECs relative to Nestin^+^ BMSC treatment alone (Additional file 6: Figure S6B, C), suggesting that the CXCL12/CXCR4 axis plays a major role in the mediating chemotactic effects observed after Nestin^+^ BMSC transplantation. As previously reported [30], recombinant human (rh)TIMP-1 enhances CXCL12-driven migration of acute myelogenous leukemia (AML) blasts in transwell migration assay in vitro, and under the presence of CXCL12 in the lower chamber, both CXCR4 antagonist (AMD3465) and anti-TIMP-1 neutralizing antibody treatment could significantly reduce the migration of AML cells. Taken together, we demonstrated that Nestin^+^BMSC-secreted TIMP-1/2 enhances CXCL12(SDF1α)/CXCR4 axis-driven migration of endogenous Sca-1^+^ CECs in ischemic heart post-AMI, and promotes the cardiac repair.

## Discussion

This study demonstrated that Nestin^+^ BMSCs show a significantly stronger capacity for tissue repair after MI than Nestin^−^ BMSCs. Nestin^+^ and Nestin^−^ cells, isolated from the compact bones of Nestin-GFP transgenic mice, both shared characteristics consistent with MSCs, as reported previously [31, 32]. An increased proliferation potential along with significantly higher chemokine levels and CEC migration was observed in Nestin^+^ BMSCs, indicating that Nestin^+^ BMSCs might be the effector cells of BMSCs in tissue repair. Notably, in vivo experiments showed that Nestin^+^ BMSCs, but not Nestin^−^ BMSCs, could restore cardiac function by recruiting CECs other than inflammatory cells, probably via the CXCL12/CXCR4 pathway. Our findings imply that Nestin^+^ BMSCs, considered to be the effector cells of BMSCs, represent a true group of MSCs and provide an ideal source of cell types for the therapy of patients with AMI. This study provides new clues for the further investigation of MSC therapy for MI in clinical trials.

Although existing treatments for MI such as coronary revascularization are conducted to restore perfusion, the therapies are usually insufficient and are associated with adverse remodeling responses. Both experimental [33, 34] and clinical studies [35, 36] have been performed concerning the extent and mechanisms of BmMSC therapy for cardiac repair, but the consequences remain controversial [13]. Given the potential promised by the use of stem cell therapy, the demand for an ideal cell type for a particular therapeutic strategy is imperative. A previous study demonstrated that compact bone was a potent ideal source for separating MSCs [31]. Thereafter, a recent study in a murine MI model evaluated the effects of the intramyocardial injection of BMSCs and showed positive results [14]. While a previous study confirmed a structural stem cell niche in bone marrow [22], it remains possible that a subpopulation of BMSCs act as the major functional cells in tissue repair.

The neural stem cell marker Nestin has been assessed in adult regeneration, where Nestin^+^ cells might act as a reservoir of progenitor cells [17, 18, 20]. Abundant evidence has verified Nestin as a marker identifying BmMSCs that participate in tissue healing [37–39]. Our study is the first to separate Nestin^+^ cells from the digested compact bones of Nestin-GFP transgenic mice using FACS. The Nestin^+^ and Nestin^−^ cells could both be expanded for months and displayed all of the characteristics of MSCs, but greater proliferation capacity was exhibited by Nestin^+^ cells. Our results verified the high progenitor potential of Nestin^+^ BMSCs, with a different source from the previous studies.

One of the major mechanisms involved in the repair process using MSCs is paracrine signaling, including growth factors, chemokines, cytokines and survival factors, all of which might mediate the repair process [11, 14, 26]. The secretion levels of paracrine factors in Nestin^+^ and Nestin^−^ BMSCs were measured, and no difference was found in the levels of growth factors. In contrast, markedly higher chemokine levels were observed in the Nestin^+^ BMSC group, while the chemotactic effects were partially blocked after the neutralization of representative chemokines, suggesting a potentially stronger chemotactic capacity. Chemotaxis, which mainly gathers immune cells to the damaged area and induces an inflammatory response, is believed to be an aggravated factor in cardiac dysfunction post-MI [27]. However, a growing number of studies have revealed the effect of chemokines on CPC recruitment in cardiac injury [40, 41]. Endogenous cardiac regeneration from resident CPCs may avoid unwanted differentiation and malignant proliferation, supporting the idea that chemokines might have therapeutic potential.

The mechanisms of therapeutic effects after MSC transplantation have been previously proposed [26]. Encouraging results in Nestin^+^ BMSC therapy, in both the survival rate and conventional echocardiography parameters, were shown in our study in vivo. As indicated in the previous results, whether chemotaxis performs a key role in restoring cardiac function warrants further study. No difference was found in the inflammatory cell infiltration among the three groups, while a marked reduction in the number of apoptotic cells was found in the Nestin^+^ BMSC group. Recent studies have shown an enhanced chemotactic effect along with a decrease in apoptotic cells in myocardium repair post-MI [42]. Thus, in this study, decreased cardiomyocyte apoptosis exhibited in the peri-ischemic myocardium might play a role in salvaging cardiac function, in addition to the enhanced chemotactic effect. Moreover, Sca-1^+^ cells were observed around the CM-Dil-labeled transplanted cells in the Nestin^+^ BMSC group. Previously, Sca-1 is a common biological marker used to identify stem cells and has been proven to be a surface marker of CPCs [28] and similar to c-kit^+^ CPCs/CSCs, which have been previously reported as the primary source for generation of new myocardium after injury [43]. However, as recently reported, c-kit^+^ cells in the adult heart are not the endogenous cardiac stem cells, which minimally contribute cardiomyocytes to the heart after injury [44], and cardiac-resident Sca-1^+^ cells are also not the CPCs but represent a subset of vascular endothelial cells that expand postnatally with enhanced responsiveness to pathological stress in vivo [45]. Our results confirmed the therapeutic effect of chemokines in Sca-1^+^CD31^+^ CEC recruitment along with cardiomyocyte salvaging instead of in inflammatory responses, a topic worthy of further consideration. The histological evidence, obtained 1 week post-MI, showed the short-term effect of the transplanted cells. Previous research has shown that CPCs can modulate the inflammatory microenvironment, further affecting cardiac regeneration [42]. Therefore, the early mobilization of CECs gathering after MI found in the Nestin^+^ BMSC group might ameliorate the microenvironment of the infarcted zone, which would be conducive to more CEC recruitment. These effects contribute to the decrease in necrotic cells, suppression of unwanted differentiation and malignant proliferation, and amelioration of the inflammatory environment, which leads to cardiac regeneration. The above evidence further indicated that Nestin^+^ BMSCs exerted their therapeutic effect via CEC recruitment.

The CXCL12 (also known as SDF-1)/CXCR4 axis has been previously reported to be involved in the retention and mobilization of stem cells in the adult ischemic cardiomyopathy, supporting the notion that CXCL12 might have therapeutic potential [46, 47]. A recent study concerning the therapeutic effect of CXCL12 delivery in an AMI model revealed the chemokine-related involvement of the recruitment of endogenous stem cells in cardiac regeneration [48]. To deeply elucidate the mechanisms underlying Nestin^+^ BMSCs’ effect on CEC recruitment, the TIMP-1, TIMP-2, and CXCL12/CXCR4 axis was blocked in this study, showing a markedly decreased LV function along with Sca-1^+^ cells. Our results verified the crucial role of the CXCL12/CXCR4 axis in the therapeutic effects of Nestin^+^ BMSC transplantation, and Nestin^+^BMSC-secreted TIMP-1/2 enhances CXCL12(SDF1α)/CXCR4 axis-driven migration of endogenous Sca-1^+^CD31^+^ CECs in ischemic heart post-AMI, and increases the cardiac function.

Therefore, our results imply that Nestin^+^ BMSCs, which were demonstrated to be the principal source of the therapeutic effects of BMSCs, could be a new subtype of seeding cells for post-MI treatment. Further experiments are warranted in large animal models, which might provide direct evidence for use in humans.

## Conclusions

Our studies indicate that Nestin^+^ BMSCs are a unique stem cell population in the compact bone that can survive the hostile environment of the post-MI heart by secreting chemokines that recruit endogenous CECs to mediate the repair progress. These improvements might provide similar effects in humans with ischemic cardiomyopathy.

## Additional files

Additional file 1: Figure S1. Differentiation capacities of bone-derived Nestin^+^ and Nestin^−^ cells in vitro. (A) Histochemical evidence of adipogenic (Oil Red O staining), osteogenic (Alizarin Red staining), and chondrogenic (Alcian Blue staining) differentiation of bone-derived Nestin^+^ and Nestin^−^ cells. Scale bar, 50 μm. (B) The qRT-PCR analysis of lineage-specific genes. The mRNA expression levels of adipocyte- (LPL, FabP4 and PPAR-γ), osteocyte- (RunX2, OSN) and chondrocyte- (collagen II and collagen X) specific markers were evaluated 14 days after differentiation induction. The means ± SEMs of the results of three different experiments are shown. **: *p* < 0.01, ***: *p* < 0.001. LPL, lipoprotein lipase; PPAR-γ, peroxisome proliferative activated receptor γ; FabP4, fatty acid binding protein 4; OSN, osteocalcin. (TIF 5292 kb)

Additional file 2: Figure S2. The protein levels of Nestin^+^ BMSCs and Nestin^−^ BMSC-derived chemokines. The protein expression of MCP-1, CXCL12, CSF-1, TIMP-1, and TIMP-2 in Nestin^+^ BMSCs, and Nestin^−^ BMSCs were analyzed by Western blots. (TIF 177 kb)

Additional file 3: Figure S3. Isolation and characteristics of cardiac Sca-1^+^ cells. (A) Flow cytometry was used to isolate Sca-1^+^ cells from hearts of postnatal day 7 C57BL/6 mice. (B) Growth curves of Sca-1^+^ cells as assessed by direct counting. Cells at P4 were seeded into a 12-well plate at 10,000 cells/well (triplicates), and the cells were then directly counted for a total of 6 days. (C) Cultured Sca-1^+^ cells expressed cardiac progenitor cell markers Sca-1, Islet-1 and cardiac transcription factors Nkx2.5, GATA4. Scale bar, 50 μm. (D) Histochemical evidence of adipogenic (Oil Red O staining) and osteogenic (Alizarin Red staining) differentiation of cardiac Sca-1^+^ cells. Scale bar, 100 μm. (E and F) The percentage of CD31 (E) and CXCR4 (F) expression in isolated Sca-1^+^ cells, which was analyzed by flow cytometry. (TIF 3252 kb)

Additional file 4: Figure S4. Isolation, characteristics and cardiac remodeling effect of bone marrow-derived Nestin^+^ cells. (A) Flow cytometry was used to isolate Nestin^+^ cells from the bone marrow of postnatal day 7 Nestin-GFP mice. (B) Histochemical evidence of adipogenic (Oil Red O staining) and osteogenic (Alizarin Red staining) differentiation of bone marrow-derived Nestin^+^ cells. Scale bar, 100 μm. (C) Flow cytometry analysis of the presence of the cell surface markers Sca-1, c-kit, CD44, CD105, CD45, CD11b on cultured bone marrow-derived Nestin^+^ cells. (D) Representative M-mode tracings from mice receiving MI + saline, MI + Nestin^+^ BMSCs or MI + Nestin^+^ BmMSCs at 1 and 4 weeks post-MI (*n* = 12 for each group). (E) Ejection fraction of different groups derived from echocardiography measurements. The means ± SEMs of the results are shown. *: *p* < 0.05. (TIF 1322 kb)

Additional file 5: Figure S5. Anti-apoptosis effects on cardiac muscle HL-1 cells of Nestin^+^ and Nestin^−^ BMSCs in vitro. (A) Annexin V/PI staining and flow cytometry was used to identify apoptotic cells in control group, H_2_O_2_ treatment group, H_2_O_2_ + Nestin^+^ or Nestin^−^ BMSC co-culture group. (B) Percentage of apoptotic HL-1 cells showed significantly reduction in both BMSC co-culture group, which was most pronounced by H_2_O_2_ + Nestin^+^ BMSCs co-culture group. **, changed significantly than H_2_O_2_ treatment group, *p* < 0.01; ***, *p* < 0.001. #, changed significantly than H_2_O_2_ + Nestin^+^ BMSC co-culture group, p < 0.05. (TIF 602 kb)

Additional file 6: Figure S6. Nestin^+^ BMSCs stimulates endogenous CECs recruiting via CXCL12/CXCR4 signaling pathway. (A) Structural and functional parameters derived from echocardiography measurements after Nestin^+^ BMSC transplantation with or without AMD3100. The means ± SEMs of the results are shown. *: *p* < 0.05, **: *p* < 0.01, ***: *p* < 0.001. (B) Immunofluorescence staining of cells positive for Sca-1 (green), CM-Dil (red) and DAPI (blue) in the post-MI myocardium after Nestin^+^ BMSC transplantation with or without AMD3100 treatment (*n* = 3 for each group). Scale bar, 50 μm. (C) Percentage of Sca-1-positive cell showing the significantly reduction of Sca-1^+^ cells in Nestin^+^ BMSC+AMD3100 group. Data are shown as the mean ± SEM from five different fields. *: *p* < 0.05, ****: *p* < 0.0001. (TIF 1763 kb)

## Abbreviations

bFGF: Basic fibroblast growth factor
BmMSCs: Bone marrow-derived stem cells
BMSCs: Bone-derived mesenchymal stem cells
CECs: Cardiac endothelial cells
CFU-F: Colony-forming unit-fibroblast
DAPI: 4′,6-Diamidino-2-phenylindole
DMEM: Dulbecco’s modified essential medium
EDTA: Ethylenediamine tetraacetic acid
EF: Ejection fraction
EGF: Epidermal growth factor
FACS: Fluorescence-activated cell sorting
FBS: Fetal bovine serum
FS: Fractional shortening
HBSS: Hanks’ balanced salt solution
HE: Hematoxylin and eosin
HSC: Hematopoietic stem cell
IGF: Insulin-like growth factor
LAD: Left anterior descending
LIF: Leukemia-inhibitory factor
LV: Left ventricular
LVEDV: Left ventricular end-diastolic volumes
LVESV: Left ventricular end-systolic volumes
MI: Myocardial infarction
MSCs: Mesenchymal stem cells
PFA: Paraformaldehyde
qRT-PCR: Quantitative real-time polymerase chain reaction
TUNEL: Transferase-mediated deoxyuridine triphosphate-biotin nick end labeling
