# Supplementary figures and images for: Bone-derived Nestin-positive mesenchymal stem cells improve cardiac function via recruiting cardiac endothelial cells after myocardial infarction
Source: Stem Cell Res Ther. 2019 Apr 27;10:127. doi: 10.1186/s13287-019-1217-x (PMC6487029; doi:10.1186/s13287-019-1217-x)

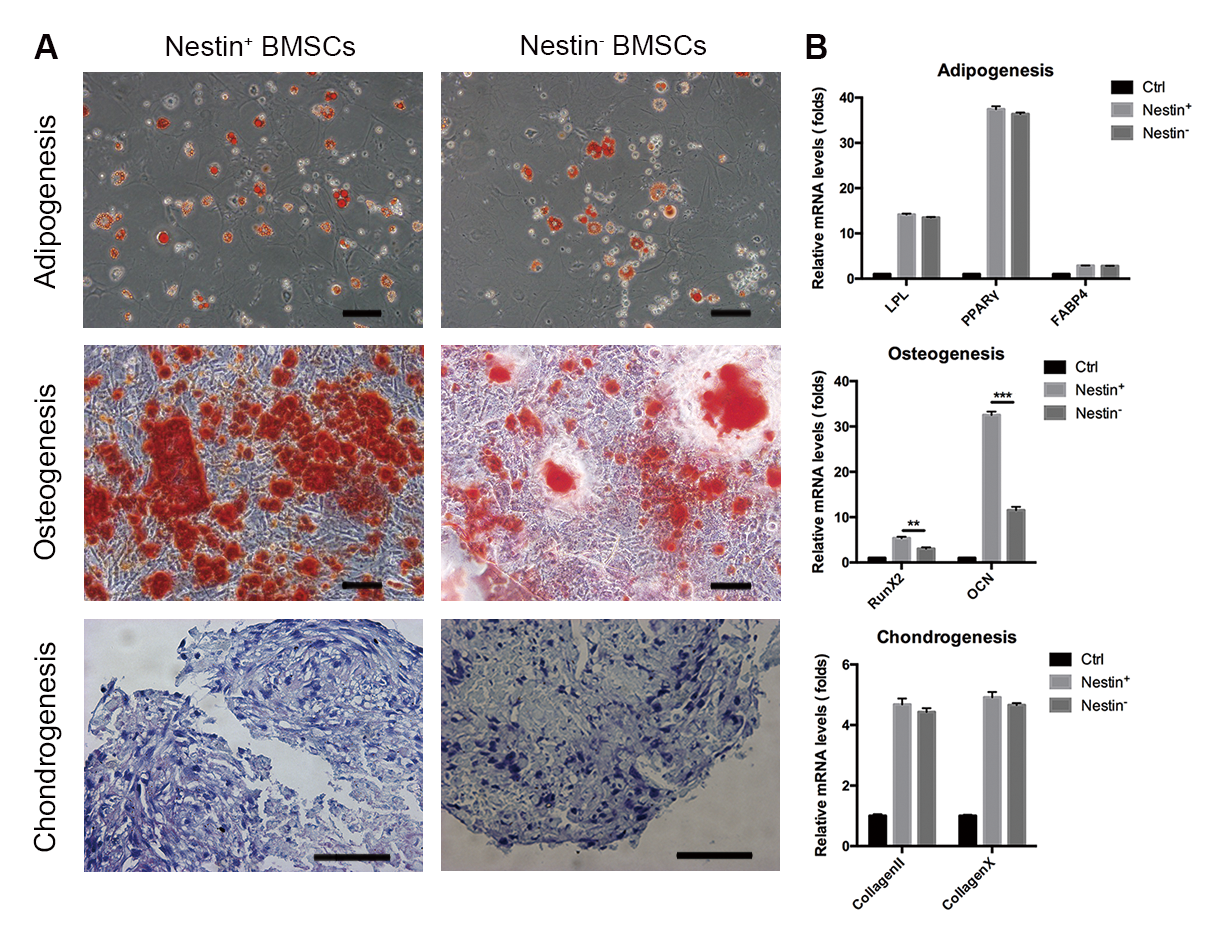

Supplement: Supplementary file 1 — Figure S1. Differentiation capacities of bone-derived Nestin+ and Nestin− cells in vitro. (A) Histochemical evidence of adipogenic (Oil Red O staining), osteogenic (Alizarin Red staining), and chondrogenic (Alcian Blue staining) differentiation of bone-derived Nestin+ and Nestin− cells. Scale bar, 50 μm. (B) The qRT-PCR analysis of lineage-specific genes. The mRNA expression levels of adipocyte- (LPL, FabP4 and PPAR-γ), osteocyte- (RunX2, OSN) and chondrocyte- (collagen II and collagen X) specific markers were evaluated 14 days after differentiation induction. The means ± SEMs of the results of three different experiments are shown. **: p < 0.01, ***: p < 0.001. LPL, lipoprotein lipase; PPAR-γ, peroxisome proliferative activated receptor γ; FabP4, fatty acid binding protein 4; OSN, osteocalcin. (TIF 5292 kb) [file 13287_2019_1217_MOESM1_ESM.tif]

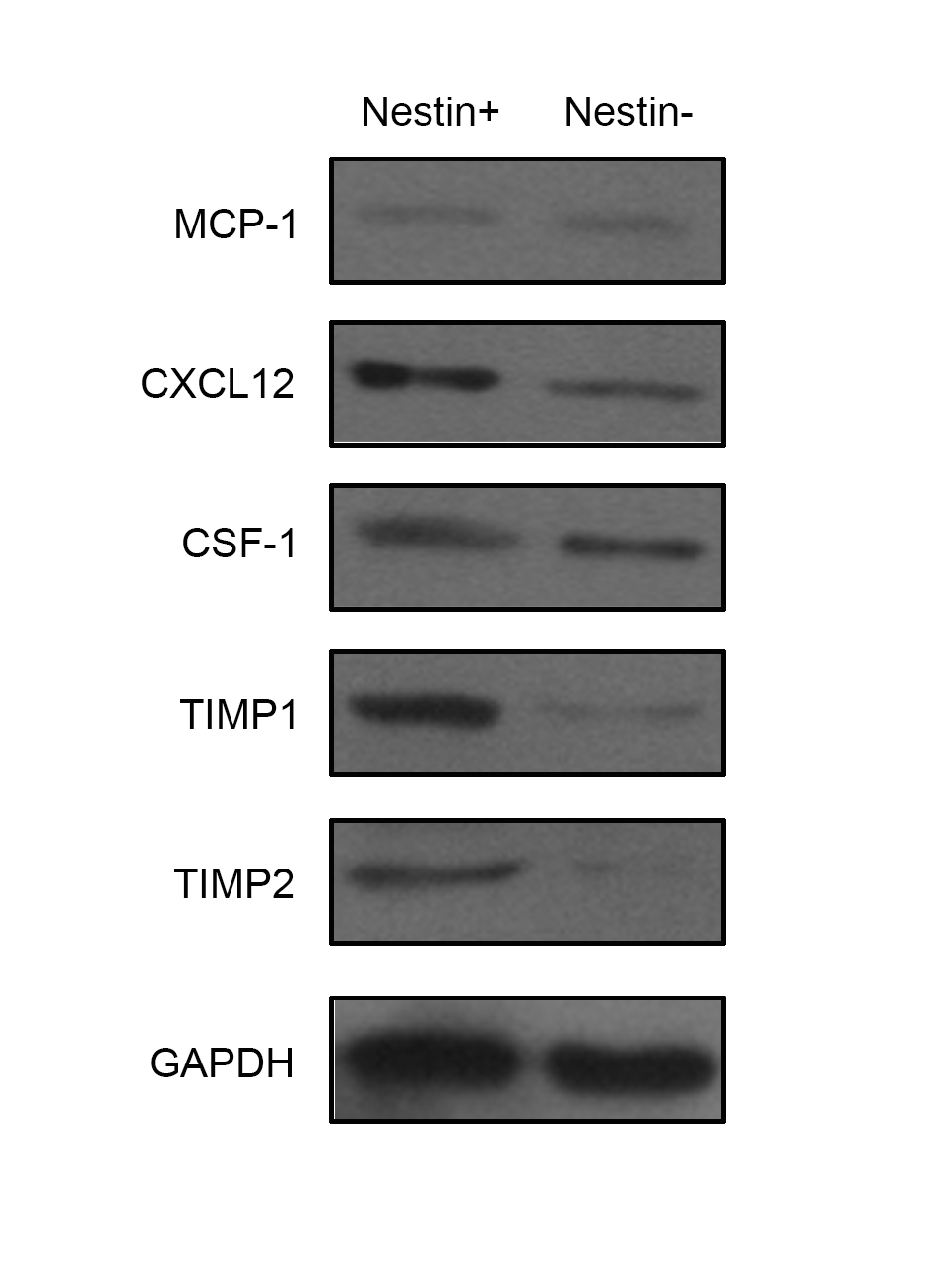

Supplement: Supplementary file 2 — Figure S2. The protein levels of Nestin+ BMSCs and Nestin− BMSC-derived chemokines. The protein expression of MCP-1, CXCL12, CSF-1, TIMP-1, and TIMP-2 in Nestin+ BMSCs, and Nestin− BMSCs were analyzed by Western blots. (TIF 177 kb) [file 13287_2019_1217_MOESM2_ESM.tif]

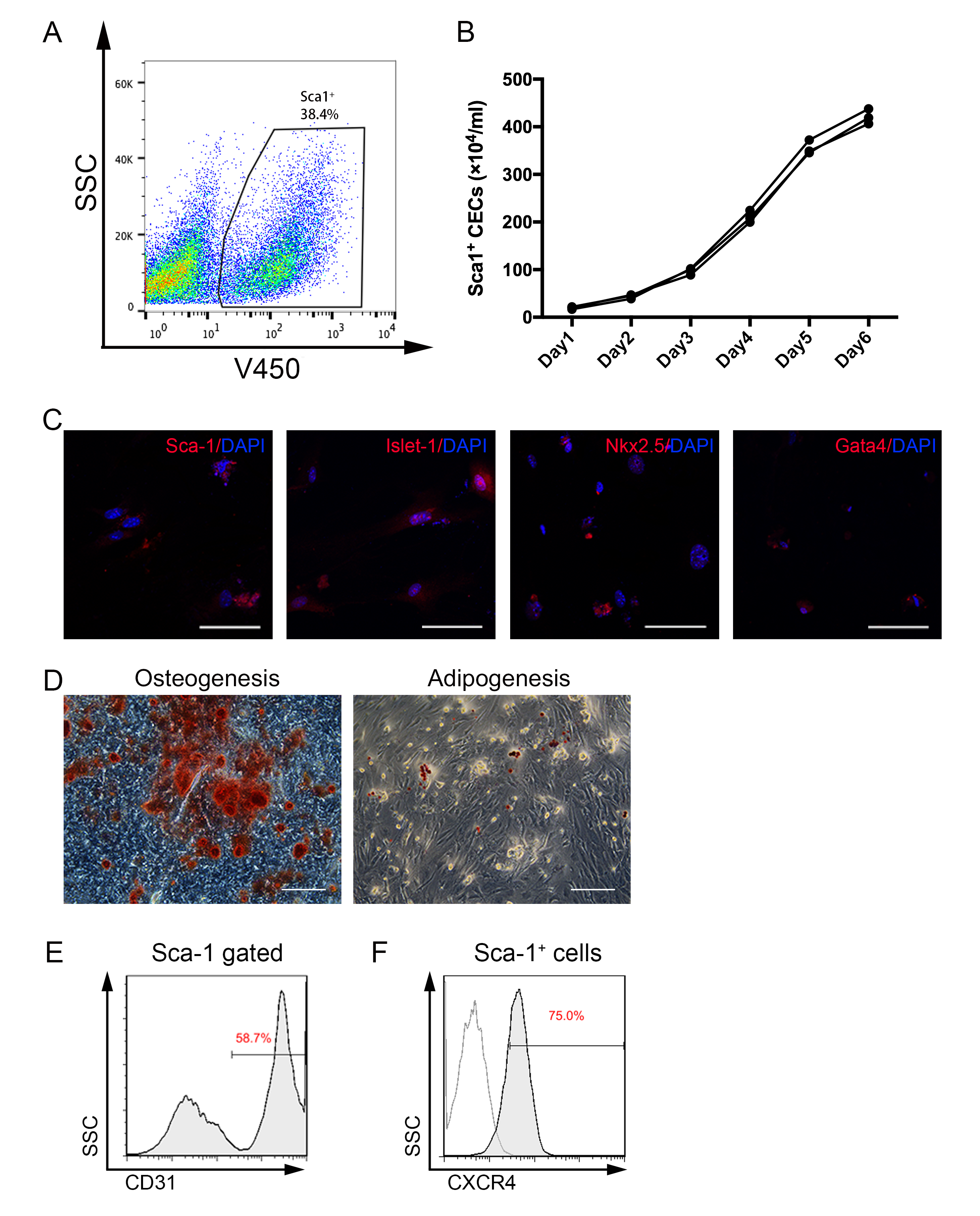

Supplement: Supplementary file 3 — Figure S3. Isolation and characteristics of cardiac Sca-1+ cells. (A) Flow cytometry was used to isolate Sca-1+ cells from hearts of postnatal day 7 C57BL/6 mice. (B) Growth curves of Sca-1+ cells as assessed by direct counting. Cells at P4 were seeded into a 12-well plate at 10,000 cells/well (triplicates), and the cells were then directly counted for a total of 6 days. (C) Cultured Sca-1+ cells expressed cardiac progenitor cell markers Sca-1, Islet-1 and cardiac transcription factors Nkx2.5, GATA4. Scale bar, 50 μm. (D) Histochemical evidence of adipogenic (Oil Red O staining) and osteogenic (Alizarin Red staining) differentiation of cardiac Sca-1+ cells. Scale bar, 100 μm. (E and F) The percentage of CD31 (E) and CXCR4 (F) expression in isolated Sca-1+ cells, which was analyzed by flow cytometry. (TIF 3252 kb) [file 13287_2019_1217_MOESM3_ESM.tif]

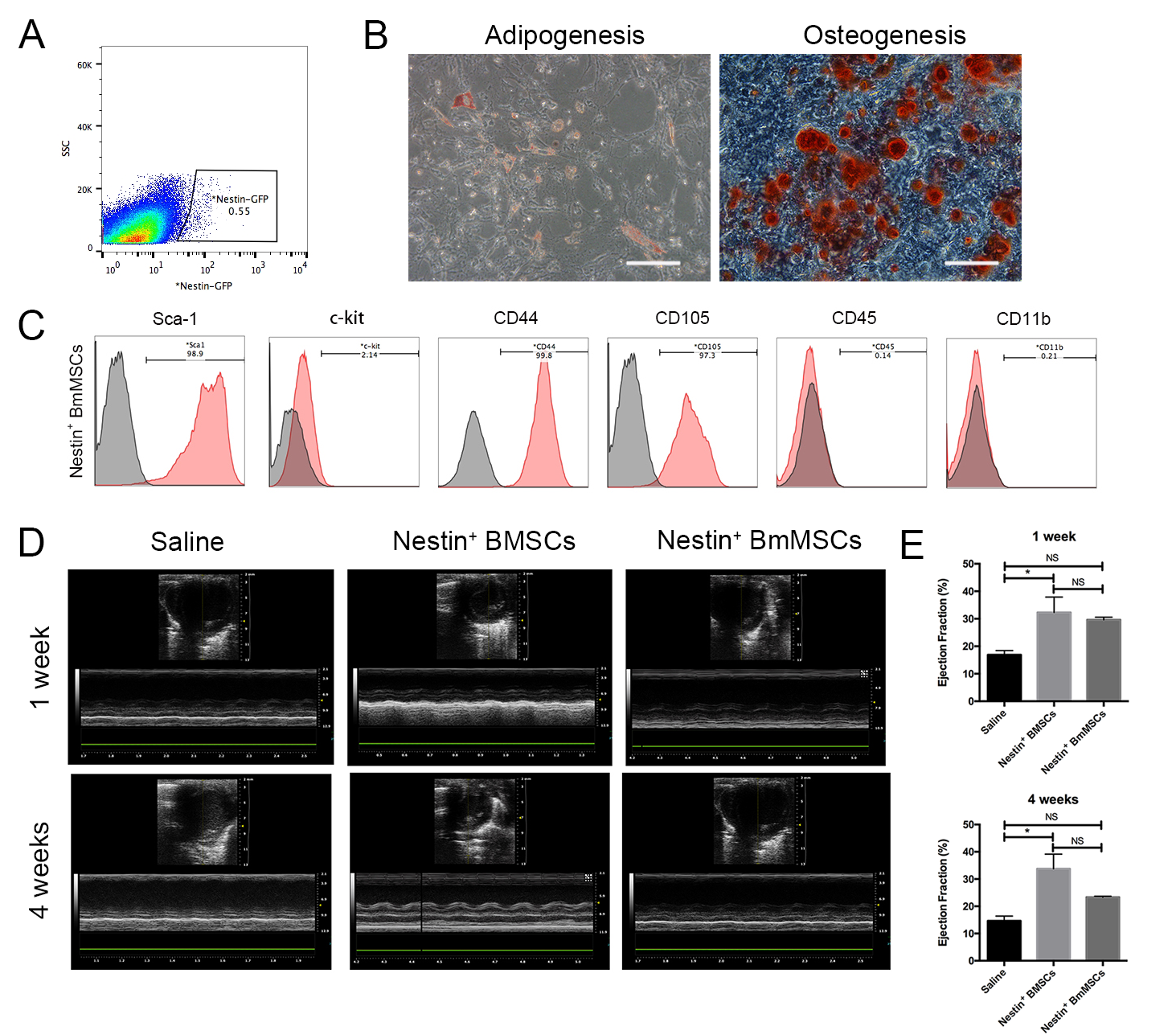

Supplement: Supplementary file 4 — Figure S4. Isolation, characteristics and cardiac remodeling effect of bone marrow-derived Nestin+ cells. (A) Flow cytometry was used to isolate Nestin+ cells from the bone marrow of postnatal day 7 Nestin-GFP mice. (B) Histochemical evidence of adipogenic (Oil Red O staining) and osteogenic (Alizarin Red staining) differentiation of bone marrow-derived Nestin+ cells. Scale bar, 100 μm. (C) Flow cytometry analysis of the presence of the cell surface markers Sca-1, c-kit, CD44, CD105, CD45, CD11b on cultured bone marrow-derived Nestin+ cells. (D) Representative M-mode tracings from mice receiving MI + saline, MI + Nestin+ BMSCs or MI + Nestin+ BmMSCs at 1 and 4 weeks post-MI (n = 12 for each group). (E) Ejection fraction of different groups derived from echocardiography measurements. The means ± SEMs of the results are shown. *: p < 0.05. (TIF 1322 kb) [file 13287_2019_1217_MOESM4_ESM.tif]

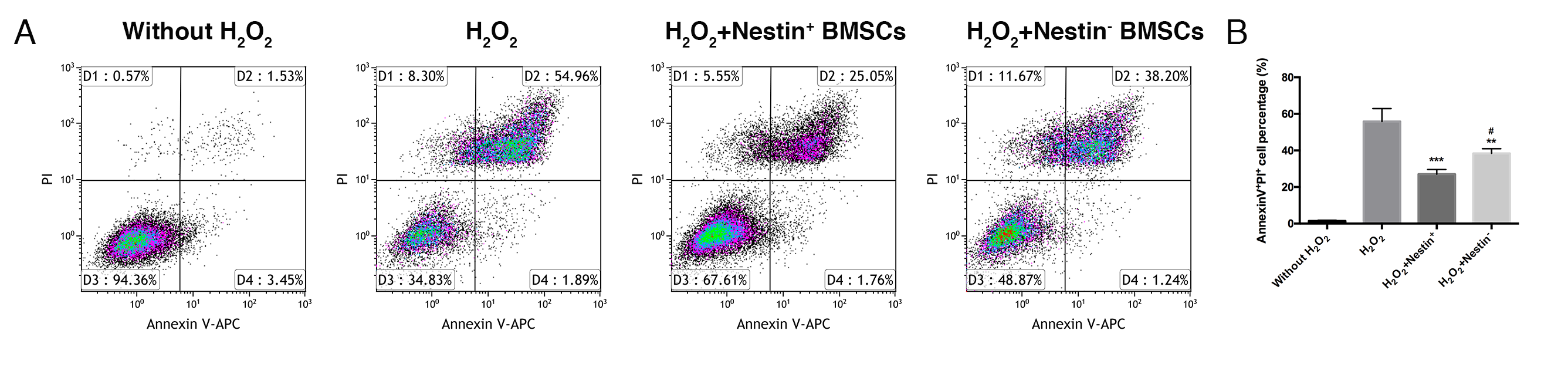

Supplement: Supplementary file 5 — Figure S5. Anti-apoptosis effects on cardiac muscle HL-1 cells of Nestin+ and Nestin− BMSCs in vitro. (A) Annexin V/PI staining and flow cytometry was used to identify apoptotic cells in control group, H2O2 treatment group, H2O2 + Nestin+ or Nestin− BMSC co-culture group. (B) Percentage of apoptotic HL-1 cells showed significantly reduction in both BMSC co-culture group, which was most pronounced by H2O2 + Nestin+ BMSCs co-culture group. **, changed significantly than H2O2 treatment group, p < 0.01; ***, p < 0.001. #, changed significantly than H2O2 + Nestin+ BMSC co-culture group, p < 0.05. (TIF 602 kb) [file 13287_2019_1217_MOESM5_ESM.tif]

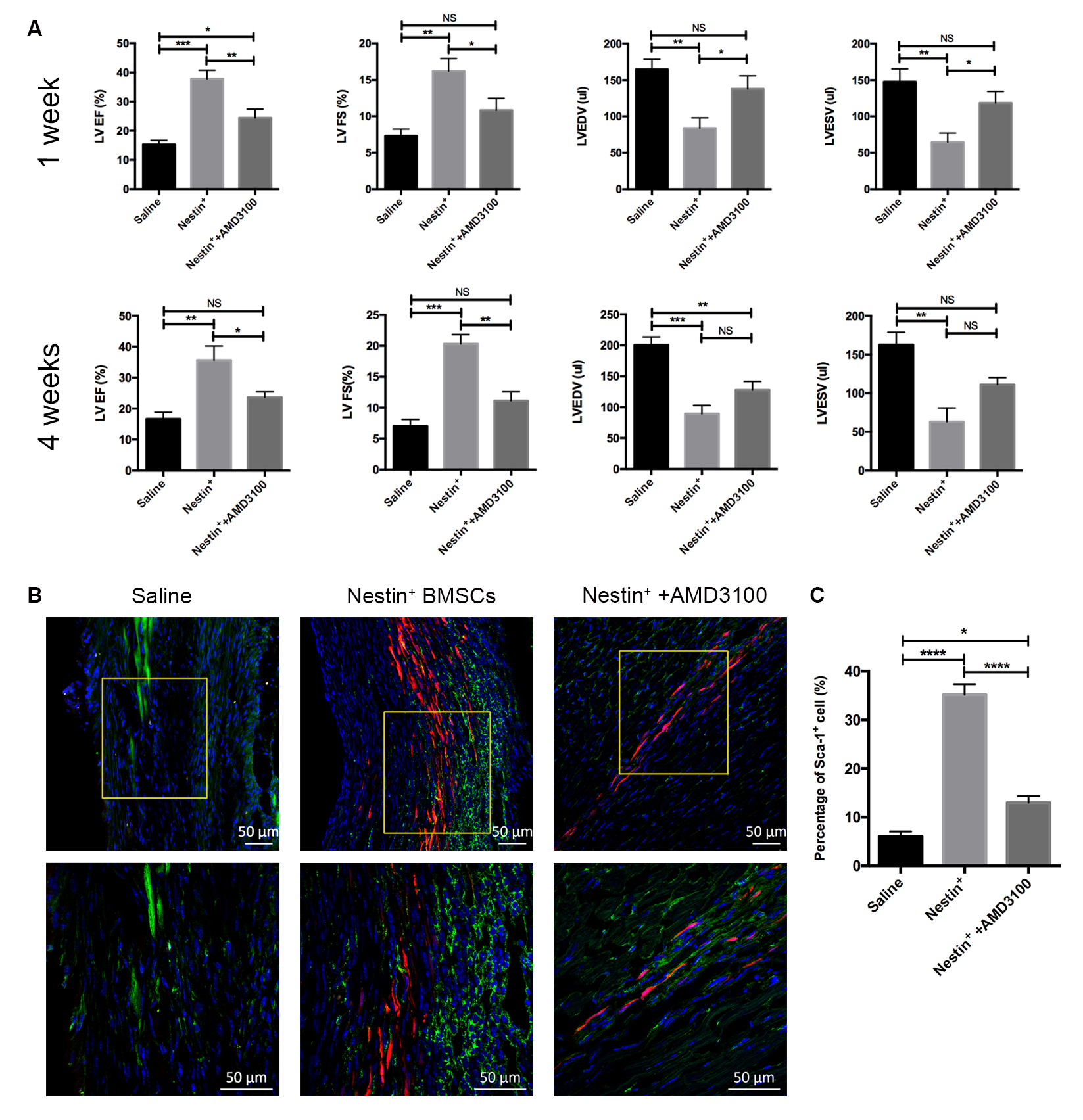

Supplement: Supplementary file 6 — Figure S6. Nestin+ BMSCs stimulates endogenous CECs recruiting via CXCL12/CXCR4 signaling pathway. (A) Structural and functional parameters derived from echocardiography measurements after Nestin+ BMSC transplantation with or without AMD3100. The means ± SEMs of the results are shown. *: p < 0.05, **: p < 0.01, ***: p < 0.001. (B) Immunofluorescence staining of cells positive for Sca-1 (green), CM-Dil (red) and DAPI (blue) in the post-MI myocardium after Nestin+ BMSC transplantation with or without AMD3100 treatment (n = 3 for each group). Scale bar, 50 μm. (C) Percentage of Sca-1-positive cell showing the significantly reduction of Sca-1+ cells in Nestin+ BMSC+AMD3100 group. Data are shown as the mean ± SEM from five different fields. *: p < 0.05, ****: p < 0.0001. (TIF 1763 kb) [file 13287_2019_1217_MOESM6_ESM.tif]
